# Case Report: Fecal microbiota transplantation resensitizes advanced non-small cell lung cancer to platinum-based chemotherapy

**DOI:** 10.3389/fonc.2026.1876788

**Published:** 2026-07-28

**Authors:** Yun Joon Lee, Jung Hwan Lee, Boram Cha, Jongbeom Shin, Kye-Sook Kwon, Yong Woon Shin, Kyung Hee Lee, Jun Hyeok Lim

**Affiliations:** 1 College of Medicine, Inha University, Incheon, Republic of Korea; 2 Division of Gastroenterology, Department of Internal Medicine, Inha University College of Medicine, Inha University Hospital, Incheon, Republic of Korea; 3 Department of Hospital Medicine, Inha University College of Medicine, Inha University Hospital, Incheon, Republic of Korea; 4 Department of Radiology, Inha University School of Medicine, Inha University Hospital, Incheon, Republic of Korea; 5 Division of Pulmonology, Department of Internal Medicine, Inha University College of Medicine, Inha University Hospital, Incheon, Republic of Korea

**Keywords:** 16S rRNA, chemotherapy, *Clostridioides difficile* infection, fecal microbiota transplantation, gut microbiota, non-small cell lung cancer

## Abstract

Platinum-based chemotherapy remains a key treatment for advanced non-small cell lung cancer (NSCLC) in patients without actionable mutations or response to immunotherapy. Emerging evidence highlights the gut–lung axis as an important factor influencing lung cancer biology and treatment outcomes. Fecal microbiota transplantation (FMT) may restore gut microbial balance and improve anticancer therapy response. However, evidence in patients receiving conventional chemotherapy is limited. We report a case of FMT used to treat refractory *Clostridioides difficile* infection (CDI) in a patient with advanced NSCLC and evaluate its effects on gut microbial reconstitution and clinical response to chemotherapy. A 64-year-old woman with heavily pretreated NSCLC developed recurrent CDI during eighth-line chemotherapy (etoposide and carboplatin). After failure of standard antibiotic therapy, she underwent colonoscopic FMT. Gut microbiota shifts were analyzed using 16S rRNA long-read sequencing (V1–V10), with taxonomic assignment based on the NCBI 16S database. Following FMT, diarrheal symptoms resolved, and the patient completed the subsequent chemotherapy cycle. Follow-up chest computed tomography showed radiologic improvement, with a reduction in the linear extent of consolidation from 91.4 to 76.7 mm. Microbiological analysis revealed a shift toward gut homeostasis, including increased abundance of *Bacillota* (formerly Firmicutes), enrichment of *Bifidobacterium breve*, and depletion of *Klebsiella pneumoniae.* This case suggests that FMT effectively treats refractory CDI in patients with advanced cancer and may support the efficacy of conventional cytotoxic chemotherapy by modulating the immunometabolic milieu through the gut–lung axis.

## 1 Introduction

Over the past several decades, first-line treatment for advanced non-small cell lung cancer (NSCLC) has shifted toward immune checkpoint inhibitors (ICIs) and targeted therapies (1, 2). Nevertheless, platinum-based chemotherapy remains a key therapeutic backbone for patients without actionable driver alterations or those who do not benefit from immunotherapy. The gut–lung axis, which refers to bidirectional cross-talk between the intestinal microbiota and the lungs, has emerged as a key concept in the pathogenesis of pulmonary diseases, including asthma, chronic obstructive pulmonary disease, infectious diseases, and lung cancer (3, 4). Fecal microbiota transplantation (FMT) has been proposed as a strategy to restore gut microbial balance and potentially enhance anticancer treatment outcomes. Although accumulating evidence supports the clinical relevance of FMT in combination with ICIs, data regarding its role in patients receiving conventional cytotoxic chemotherapy remain limited (5). Here, we report a case of a heavily pretreated patient with advanced NSCLC who underwent FMT for refractory *Clostridioides difficile* infection during ongoing chemotherapy, followed by radiologic improvement.

## 2 Case description

**Figure 1:**
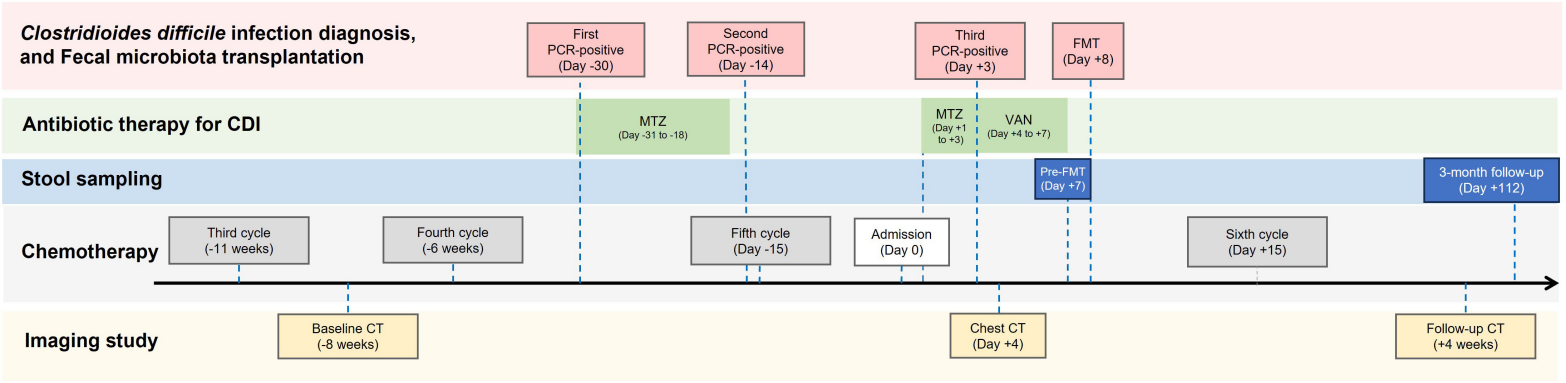
Clinical timeline of *Clostridioides difficile* infection (CDI), antibiotic therapy, fecal microbiota transplantation (FMT), stool sampling, chemotherapy, and imaging studies. All events are presented relative to the date of hospital admission (Day 0). PCR, polymerase chain reaction; CDI, *C. difficile* infection; MTZ, metronidazole; VAN, vancomycin; CT, computed tomography.

**Figure 2:**
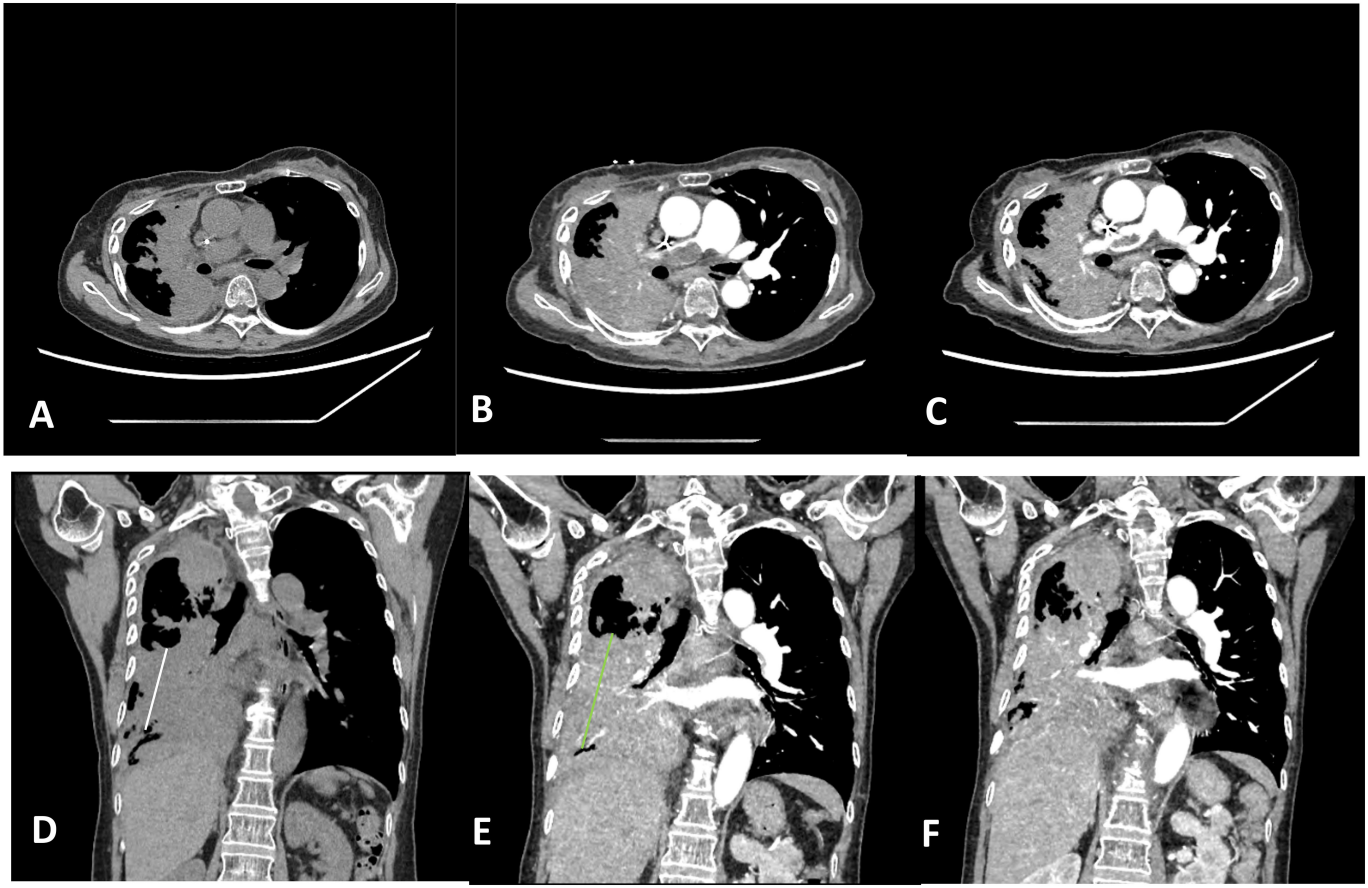
Chest computed tomography (CT) scans obtained at baseline and before and after fecal microbiota transplantation and the sixth cycle of etoposide and carboplatin chemotherapy. **(A)** Baseline chest CT shows near-total consolidation of the right lung with partially aerated areas in the right upper lobe. **(B)** Chest CT before fecal microbiota transplantation shows increased consolidation involving the right lower lobe. **(C)** Follow-up chest CT after fecal microbiota transplantation and the sixth cycle of chemotherapy shows newly developed aeration in the right lower lobe. **(D–F)** The linear extent of consolidation was measured on the same coronal slice and is indicated by white, green, and yellow lines (**(D)**: 68.6 mm; **(E)**: 91.4 mm; and **(F)**: 76.7 mm, respectively).

A 64-year-old woman with a history of NSCLC was admitted with generalized weakness and persistent diarrhea. She had been diagnosed with adenocarcinoma (T2N3M0) 6 years earlier and had undergone multiple lines of systemic therapy, including afatinib, gemcitabine plus cisplatin, pembrolizumab, pemetrexed, vinorelbine, docetaxel, and irinotecan plus cisplatin; all were discontinued owing to disease progression. Two weeks prior to the current admission, the patient had completed the fifth cycle of etoposide and carboplatin combination chemotherapy as eighth-line systemic therapy. The clinical timeline is shown in **Figure 1**. Post–third-cycle evaluation demonstrated partial aeration of the right upper and lower lobes, with near-total consolidation and nodules in the right lung (**Figures 2A, D**). Brain magnetic resonance imaging showed no intracranial metastatic lesions, and fluorodeoxyglucose positron emission tomography revealed hypermetabolic lesions in both lungs with hypermetabolic right hilar and mediastinal lymph nodes, as well as multiple skeletal lesions suspicious for metastases.

The patient’s vital signs were as follows: blood pressure, 100/70 mmHg; heart rate, 110 beats per minute; respiratory rate, 18 breaths per minute; and body temperature, 37.7 °C. Laboratory evaluation revealed a white blood cell count of 4,350 cells/µL, C-reactive protein level of 15.57 mg/dL, and erythrocyte sedimentation rate of 89 mm/h.

One month before admission, the patient was hospitalized for diarrhea and diagnosed with *C. difficile* infection (CDI) based on a positive *C. difficile* polymerase chain reaction (PCR) test. She was subsequently treated with oral metronidazole (500 mg, three times daily for 14 days); however, diarrhea recurred and persisted. Based on a positive *C. difficile* PCR assay on admission, the patient was diagnosed with recurrent CDI. Treatment with oral metronidazole (500 mg, three times daily for 3 days) and oral vancomycin (125 mg, four times daily for 4 days) was initiated. Concurrently, the patient received intravenous piperacillin/tazobactam from hospital days 2 to 10 for empirical coverage of potential secondary infections.

On hospital day 4, computed tomography (CT) of the chest and lower extremities was performed to evaluate newly developed lower extremity edema. CT revealed pulmonary thromboembolism (PTE) in the right pulmonary artery (**Figure 2B**). Concomitant deep vein thrombosis involving the infrarenal inferior vena cava was also observed. Compared with imaging obtained two months earlier, the right lung mass demonstrated a slight interval enlargement (**Figures 2B, E**). The patient underwent inferior vena cava filter insertion for the management of PTE and deep vein thrombosis. The patient underwent intravenous heparinization for 1 week alongside compression stocking application, which was subsequently transitioned to oral rivaroxaban (15 mg, twice daily).

Despite appropriate antibiotic therapy for recurrent CDI, the patient continued to experience frequent loose stools. Given the refractory nature of CDI, FMT was performed on hospital day 9 using stool from a healthy donor. Approximately 300 mL of prepared fecal suspension was instilled throughout the colon via colonoscopy. Stool samples for microbiological analysis were collected 1 day before and 105 days (3 months) after FMT. To characterize the microbial community, full-length 16S rRNA gene sequencing was performed using the PacBio 16S full-length Library kit and the PacBio Revio system. Sequencing generated 72,451 to 91,843 high-fidelity reads per sample (≥99% predicted accuracy), with average read lengths of 1,486–1,515 bp and mean quality scores ranging from Q37 to Q39. For bioinformatics processing, primer sequences were removed using Cutadapt (v3.2), followed by sequence denoising, quality filtering (1,000–1,600 bp), and exact amplicon sequence variant (ASV) inference using DADA2 (v1.18.0) optimized for long-read data (6, 7). Taxonomic assignment of the finalized ASVs was performed using the DADA2 naive Bayesian classifier against the NCBI_16S database (8). Following multiple sequence alignment and phylogenetic tree construction via Mafft (v7.475) and FastTreeMP (v2.1.10), downstream microbial diversity analyses were conducted using QIIME (v1.9.0) (9, 10). Microbial community structure was statistically validated using alpha-diversity indices (Chao1, Shannon, and Simpson) and beta-diversity distances (weighted and unweighted UniFrac matrices).

Following FMT, the patient’s diarrhea markedly improved. On hospital day 18, she received the planned sixth cycle of etoposide and carboplatin chemotherapy without significant adverse events and was subsequently discharged in a stable condition.

Chest CT was repeated for follow-up evaluation of PTE. Follow-up CT demonstrated improvement in dependent consolidation and aeration of the right lower lobe compared with the prior chest CT (**Figures 2C, F**).

However, the patient’s post-discharge clinical course was undermined by subsequent hemorrhagic complications. Three weeks after FMT, she developed gross hemoptysis, which required urgent readmission and the immediate withholding of rivaroxaban. Once the respiratory bleeding was successfully controlled, oral anticoagulation was cautiously resumed using apixaban (5 mg, twice daily). Unfortunately, 10 weeks post FMT, the patient suffered severe gastrointestinal bleeding, necessitating endoscopic hemostasis. These recurrent bleeding episodes forced the permanent cessation of all systemic cytotoxic regimens. Without further therapeutic intervention, the tumor progressed, and the patient died 6 months after FMT.

## 3 Discussion

We describe a heavily pretreated patient with advanced NSCLC who exhibited clinical stabilization and radiologic improvement after FMT for refractory CDI during ongoing etoposide and carboplatin chemotherapy.

The gut microbiota is increasingly recognized as a key regulator of oncologic outcomes, with dysbiosis implicated in carcinogenesis as well as in the modulation of treatment response, efficacy, and toxicity (11). Although substantial clinical evidence suggests that FMT may enhance responses to ICIs, particularly anti–PD-1 therapy, its specific role in conventional cytotoxic chemotherapy remains poorly understood (5).

**Figure 3:**
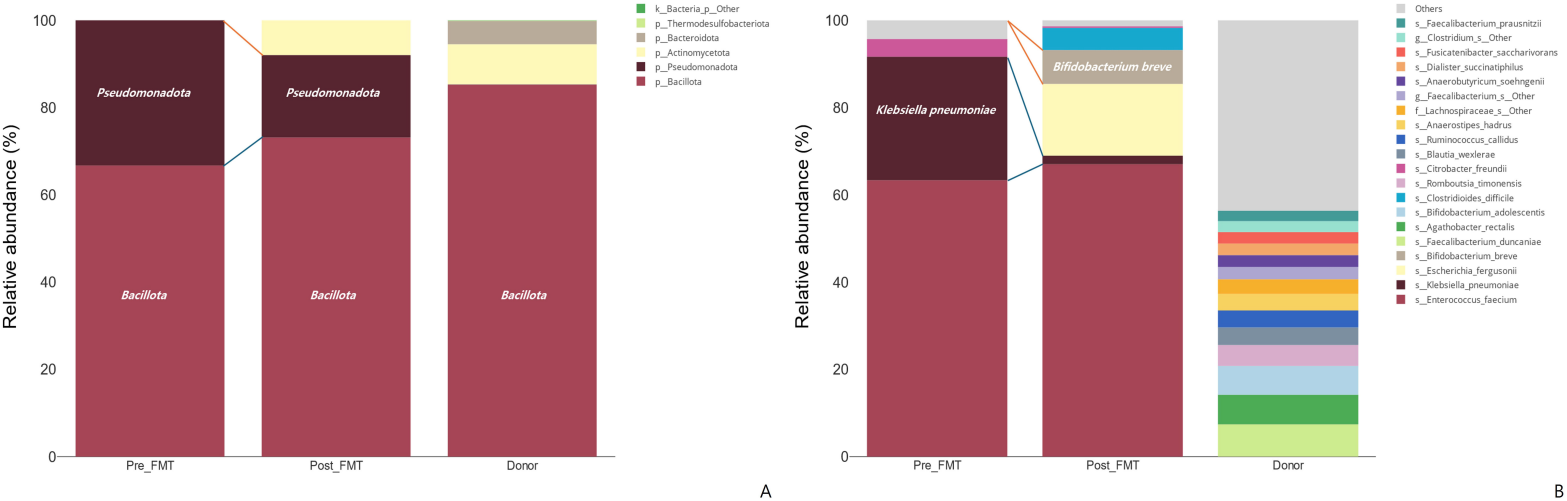
Gut microbiota composition before and after fecal microbiota transplantation (FMT). **(A)** Phylum-level relative abundance shows an increase in *Bacillota* and a decrease in *Pseudomonadota* post-FMT. **(B)** Species-level analysis demonstrates successful enrichment of beneficial *Bifidobacterium breve* and depletion of pathogenic *Klebsiella pneumoniae*, facilitating a favorable therapeutic response to chemotherapy. Gut microbiota were analyzed using 16S rRNA long-read sequencing (V1–V10), with taxonomic assignment performed by matching sequences against the NCBI 16S database.

In this context, our case suggests that FMT-induced restoration of gut microbial diversity may contribute to improved chemotherapy responsiveness through modulation of the gut–lung axis (**Figure 3**). Notably, post-FMT microbial shifts, including the expansion of beneficial taxa such as *Bifidobacterium breve* and the marked reduction of pathogenic species such as *Klebsiella pneumoniae*, indicate a transition toward a more favorable immunometabolic milieu (**Figure 3A**). These findings are consistent with those of previous studies demonstrating that specific microbial communities can augment antitumor immunity and influence chemotherapy outcomes by affecting systemic inflammation, host immune activation, and drug metabolism (12, 13).

Mechanistically, the observed microbiome reconstitution may have enhanced anticancer effects through multiple complementary pathways. The emergence of *B. breve* is notable given its reported role in promoting dendritic cell activation, IL-12 production, and CD8+ T-cell–mediated antitumor immunity, as well as enhancing apoptosis in tumor cells via caspase-dependent mechanisms (12, 14–16). Concurrently, the reduction in *K. pneumoniae* may mitigate immunosuppressive signaling and reduce microbial-mediated drug inactivation, thereby improving chemotherapeutic efficacy (17). Furthermore, the increased abundance of *Bacillota* suggests enhanced production of short-chain fatty acids such as butyrate, which support epithelial integrity and sustain antitumor immune responses via the metabolic and epigenetic reprogramming of T cells (**Figure 3B**) (18, 19).

In our case, the patient’s gut microbiota was likely severely disrupted by multiple previous lines of systemic therapy and recurrent CDI. FMT may have restored microbial diversity, thereby enhancing the therapeutic effect of the subsequent chemotherapy cycle. An alternative explanation for the observed radiologic improvement is a delayed response to chemotherapy. However, unlike immunotherapies, conventional cytotoxic regimens typically achieve maximum objective responses early in the treatment course (within 2–3 cycles), and delayed tumor shrinkage after multiple cycles of progressive disease is clinically highly improbable (20, 21). In our patient, disease progression had been documented throughout multiple prior treatment lines, and a slight interval enlargement of the lung lesion was explicitly observed after the fifth etoposide/carboplatin cycle, indicating resistance to the ongoing regimen prior to FMT. Furthermore, the efficacy of later-line chemotherapy in advanced NSCLC is generally limited, with a reported median progression-free survival of 51 days and a disease control rate of 39% after third-line treatment (22). Although causality cannot be established, the radiologic improvement observed only after FMT indicates that microbiota restoration may have contributed to renewed chemotherapy responsiveness. Alpha-diversity metrics also increased after FMT (**Table 1**), indicating restoration of microbial richness, evenness, and phylogenetic diversity.

**Table 1:** Alpha-diversity analysis of gut microbiota before and after fecal microbiota transplantation (FMT).

Sample	ASV	Chao1	Shannon	Faith's PD	Simpson	Gini-Simpson
Pre-FMT	32	32	3.50	1.30	0.15	0.85
Post-FMT (3-month follow-up)	41	41	4.06	2.10	0.08	0.92

ASV, amplicon sequence variant; Faith's PD, Faith's phylogenetic diversity. Higher values of ASV, Chao1, Shannon, Faith's PD, and Gini-Simpson indicate greater microbial richness and diversity, whereas lower Simpson values indicate reduced dominance by individual taxa.

Furthermore, the patient received multiple concurrent clinical interventions that may confound the interpretation of the treatment outcome. During hospitalization, the administration of piperacillin/tazobactam profoundly disrupted the gut microbiota, as this broad-spectrum agent thoroughly depletes beneficial short-chain fatty acid-producing genera, such as *Blautia* and *Lactobacillus*, and drastically reduces alpha-diversity (23, 24). This antibiotic-induced collapse of the commensal barrier facilitates a permissive environment for the opportunistic overgrowth of *K. pneumoniae*, as evident in our pre-FMT profiling (25). Remarkably, FMT successfully inverted this hostile microenvironment, suppressing the pathogen and reinstating compositional homeostasis.

The initiation of anticoagulation with rivaroxaban for acute thromboembolism presents another critical confounder. Mechanistically, rivaroxaban blocks factor Xa and downregulates downstream protease-activated receptor 2 signaling, a pathway hypothesized to enhance responsiveness to both immunotherapy and cytotoxic chemotherapy (26). Although long-term anticoagulation has been associated with significantly lower cancer-related mortality in select cohorts, our patient received this therapy for less than 1 month (27). The clinical course was rapidly complicated by severe hemoptysis and gastrointestinal bleeding, ultimately resulting in a poor prognosis; these hemorrhagic events forced the permanent discontinuation of all cytotoxic chemotherapy after the sixth etoposide/carboplatin cycle, and the patient succumbed to disease progression 6 months post-FMT without further systemic therapy. Therefore, while the final outcome was heavily driven by these severe clinical complications, the radiologic improvement following the reversal of an antibiotic-extinguished gut milieu suggests that FMT holds a scientifically plausible, albeit hypothesis-generating, potential to support the efficacy of conventional cytotoxic chemotherapy.

Supporting this interpretation, preclinical studies have demonstrated that gut microbiota depletion impairs chemotherapy-induced reactive oxygen species generation and antitumor cytotoxicity, whereas microbiota restoration, such as through FMT, can reverse these effects (13, 28). In our patient, repeated prior therapies and recurrent infections likely resulted in profound dysbiosis, and the simultaneous enrichment of beneficial taxa (i.e., *B. breve*) and the depletion of pathogenic organisms (i.e., *K. pneumoniae*) may have synergistically contributed to the restoration of chemotherapy responsiveness, which was likely attenuated by dysbiosis.

In accordance with standard clinical reporting, we carefully expanded our diagnostic reasoning regarding the differential diagnosis of the post-FMT radiologic improvement, specifically distinguishing between a true reduction in malignant tumor mass and the mere resolution of secondary obstructive pneumonitis or atelectasis. While secondary inflammatory clearing and re-aeration routinely contribute to changes in lung consolidation, a detailed segment-by-segment radiological review strongly supported a genuine regression of the viable tumor volume. Importantly, tracing the vascular structures and mediastinal margins across the serial CT scans revealed that the dense, solid soft-tissue mass that severely encased and compressed the central pulmonary vasculature and right main bronchus at baseline and pre-FMT (**Figures 2B, E**) was significantly smaller on post-FMT follow-up images (**Figures 2C, F**). The distinct restoration of perivascular spaces and the definitive release of the solid tumor’s mass effect around major pulmonary vessels provide clear morphological evidence of core tumor mass shrinkage. This shrinkage cannot be explained solely by the clearing of secondary dependent pneumonia or re-inflation of collapsed alveoli. Although the resolution of downstream obstructive atelectasis aided in improving peripheral lung aeration, the concurrent structural regression of the central invasive mass confirms a true anti-tumor response, underpinning that microbiota restoration may have contributed to renewed chemotherapy responsiveness.

This report is limited by its nature as a single case study. Despite the limitation, the favorable clinical response and stabilization observed in our patient suggest that FMT could be strategically integrated into conventional chemotherapy regimens to optimize treatment outcomes in advanced NSCLC.

## Data Availability

The original contributions presented in the study are included in the article. Further inquiries can be directed to the corresponding authors.
